# Inverse association of a traditional Korean diet composed of a multigrain rice-containing meal with fruits and nuts with metabolic syndrome risk: The KoGES

**DOI:** 10.3389/fnut.2022.1051637

**Published:** 2022-11-10

**Authors:** Min Jung Kim, Haeng Jeon Hur, Dai Ja Jang, Myung-Sunny Kim, Sunmin Park, Hye Jeong Yang

**Affiliations:** ^1^Food Functionality Research Division, Korea Food Research Institute, Seongnam-si, South Korea; ^2^Department of Food Biotechnology, University of Science and Technology, Wanju, South Korea; ^3^Department of Food and Nutrition, Obesity/Diabetes Research Center, Hoseo University, Asan-si, South Korea; ^4^R&D, Yejunbio, Asan-si, South Korea

**Keywords:** traditional Korean diet, hansik, K_diet_-index, metabolic syndrome, abdominal obesity, hyperglycemia, dyslipidemia, skeletal muscle mass

## Abstract

**Background:**

Hansik, a traditional Korean diet, may have a beneficial impact on metabolic syndrome (MetS) risk as dietary westernization increases its prevalence. We examined the hypothesis that adherence to the hansik diet may be inversely associated with the risk of MetS and its components and sought to understand the gender differences in 58,701 men and women aged over 40.

**Materials and methods:**

Hansik was defined using 14 components from which the Korean dietary pattern index (K_diet_-index) was generated by summing their scores. Low-hansik intake was defined as the K_diet_-index with <8. MetS was categorized based on the 2005 revised NCEP-ATP III criteria modified for Asians.

**Results:**

The K_diet_-index score was negatively associated with the dietary inflammation index and showed that the high intake of a meal with multigrain rice, fruits, and their products, and nuts, and low intake of fried foods were inversely associated with MetS by 0.707, 0.864, 0.769, and 0.918 times, respectively, after adjusting for covariates. More women and participants with more educated and lower income belonged to the high-hansik group, and participants with high self-rated health scores consumed more hansik. All participants on a high-hansik diet were associated with a 0.87 time lower risk of MetS. Specifically, the association between hansik intake and MetS risk was not significant among men following stratification by gender. Body composition, including the body mass index, waist circumference, and fat mass, was inversely associated with hansik intake, while the skeletal muscle mass index was positively associated with the hansik intake in each gender and all participants. In all the participants in the high-hansik group, no significant changes were seen in the serum glucose and HDL concentration. However, a high-hansik intake showed lower blood pressure and serum LDL and triglyceride concentrations only in men and a higher glomerular filtration rate in both genders.

**Conclusions:**

Hansik intake might improve MetS risk, with its primary beneficial effects on body composition, dyslipidemia, and blood pressure gender-dependently.

## Introduction

Metabolic syndrome (MetS) is a cluster of several physiological and metabolic abnormalities that raise the risk of atherosclerotic cardiovascular disease, insulin resistance, and type 2 diabetes. A diagnosis of metabolic syndrome is made when a patient presents with three or more of the following: abdominal obesity, hyperglycemia, hypertriglyceridemia, reduced high-density lipoprotein cholesterol (HDL), and hypertension. The overall prevalence of MetS has risen worldwide, with different rates of prevalence in various countries (1). Gender-specific differences in the prevalence of MetS and its components have also been reported (2). In the United States (US), the MetS prevalence has increased in adults aged over 20 years from 32.5 to 36.9% over the period 2011–2012 to 2015–2016, according to the National Health and Nutrition Examination Survey (NHANES) (3). A significant increment in the 20–39 year age group, women, Asians, and Hispanics was seen (3). However, in Korea, the MetS prevalence in men increased from 24.5 to 28.1% between 2008 and 2017, while no significant increase was seen among women (20.5 and 20.7%), according to the Korea National Health and Nutrition Examination Survey (KNHANES) (4). Therefore, the differences in the MetS prevalence among Asian women in the US and Korea may be related to various lifestyle factors, especially dietary patterns (5). Scientists have made efforts to identify a dietary pattern that is easy to follow to prevent and alleviate MetS risk, such as the Mediterranean diet and Dietary Approaches to Stop Hypertension (DASH) (6). However, it is challenging to incorporate these diets into Asian dietary patterns. Hence, there is a need for a country-specific beneficial dietary pattern to alleviate the rising risk of MetS.

Koreans have traditionally consumed a Korean-style diet called “hansik (K-diet; 
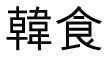
).” The basic meal of hansik includes cooked multigrain rice (a mixture of cooked rice and other grains such as brown rice, beans, barley, oat, or other grains; jabgogbab; 
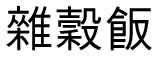
), soup made of fermented soybeans, braised or grilled fish, two servings of seasoned, cooked, or raw vegetable dishes with sesame or perilla oil (namul or saengchae, respectively), and fermented cabbage (kimchi) (7). Its basic meal pattern is called three cheopbansang (
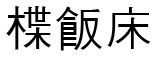
), which includes three dishes in addition to multigrain rice, soup, and kimchi. The soups contain various vegetables and small amounts of meat. A variety of vegetables are used for making namul. Grilled, baked, or stirred meats are also served instead of fish in the meal (7). Koreans ordinarily consume 3–5 cheopbansang each meal, and after each meal, fruits, nuts, and tea are served as desserts. Although the table setting is similar in every meal, the variety of vegetables used in namul and soups and the different meats and fish makes the meal nutritionally complete and healthy. Thus, the three cheopbansang in hansik can provide nutrients for meeting the dietary reference intake. The dietary pattern is easy to adhere to and provides Koreans with a balanced diet, including diverse foods, and could also be propagated to other Asian countries. Traditionally, Koreans do not consume milk and milk products that are not included in the hansik. However, their consumption as snacks has been recommended for over 50 years.

The diet pattern consumed by an individual is a crucial risk factor for the development of MetS. However, individuals find it difficult to adhere to particular foods and diets. Dietary patterns have been studied over the last decade to explore ways to reduce MetS risk while ensuring adherence. Korean-balanced diets have been shown to have an inverse association with MetS risk in randomized clinical trials (RCT) and observational studies (8–10). RCT studies are limited in the aspects of intervention periods and diet types to show the effects of traditional Korean diets. Korean food intake has been categorized into 3–4 clusters by the principal component analysis (PCA) of observational studies, which included a Korean-balanced diet. However, hansik, a traditional Korean diet, was somewhat different from the Korean-balanced diet in which multigrain, fermented soybeans, fruits, and nuts were excluded as the primary food items (11). However, the traditional Korean dietary pattern has not been studied for the association with MetS risk. In the present study, we defined the traditional Korean dietary pattern as the K_diet_-index score to show its efficacy quantitively. Therefore, we hypothesized that adherence to the hansik diet might be inversely related to the risk of MetS and its components. We also analyzed the gender-based differences of the hansik diet on MetS risk. The hypothesis was examined in a large city hospital-based cohort in Korea. Hansik intake of the participants was evaluated with 14 components to describe a traditional Korean diet, which was scored to make the K_diet_-index. The association of hansik with MetS and its components was examined using the K_diet_-index in the present study.

## Materials and methods

### Participants

The urban hospital-based cohort, a multi-institution hospital-based registry, was part of the Korean Genome and Epidemiology Study (KoGES) conducted by the Korean National Research Institute of Health (NIH), Centers for Disease Control and Prevention and the Ministry of Health and Welfare in Korea. The purpose of this cohort was to attempt to resolve the public health issues about increasing metabolic diseases related to lifestyle changes (12). Volunteers who visited the general hospitals in the metropolitan area and cities were recruited, and the inclusion criteria were aged ≥40 years at baseline. All participants (*n* = 58,701; 20,293; men and 38,408 women) in the cohort were included for the present study (12). The study was approved by the Institutional Review Board (IRB) of the National Institute of Health, Korea (KBP-2015-055) and Hoseo University (HR-034-01). The participants signed a written informed consent form.

### Anthropometric and biochemical measurements

Basic measurements such as height, weight, waist, and hip circumference were taken (13). The body mass index (BMI) was calculated by dividing the body weight (kg) by height squared (m^2^) measured while wearing light clothes and bare feet. Body fat and skeletal muscle masses were determined using the Inbody 3.0 (Cheonan, Korea) based on the Bioelectrical impedance analysis in Ansan/Ansung cohort. However, they were estimated in the city hospital-based cohort using a machine learning prediction model generated from the Ansan/Ansung cohort (14). Skeletal muscle index (SMI) was defined as dividing appendicular skeletal muscle mass by height. A doctor measured blood pressure in the left arm in a sitting position with a sphygmomanometer. Blood was collected in vacuum blood collection tubes with and without ethylenediaminetetraacetic acid (EDTA) after participants fasted overnight, and plasma and serum samples were separated for biochemical analysis. In fasting serum or plasma, biochemical parameters [glucose, total cholesterol, HDL cholesterol, triglyceride, and creatinine concentrations and aspartate aminotransferase (AST) and alanine aminotransferase (ALT) activities] were assayed using a Hitachi 7,600 automatic analyzer (Hitachi, Tokyo, Japan). Blood HbA1c contents were determined with an HbA1c Analyzer from EKF Diagnostics (Manchester, UK). Insulin resistance was calculated using Homeostatic Model Assessment for Insulin Resistance (HOMA-IR) equation by fasting serum glucose (mg/dL) and insulin (mU/L) concentrations divided by 405 (15). Serum LDL cholesterol concentrations were calculated with the Friedewald equation, excluding serum triglyceride concentrations ≥500 mg/dL. Serum high-sensitive C-reactive protein (hs-CRP) concentrations were determined with an enzyme-linked immunoassay (ELISA) kit (R&D Systems, Minneapolis, MN, USA). Exercise levels were determined based on answers to a question on exercise intensity and duration: The participants in the high-exercise group did more than three sessions per week of moderate exercise (brisk walking, mowing, badminton, swimming, tennis, and others) for >30 min or intensive exercise (climbing, running, football, basketball, volleyball, and others) for >20 min per session. The participants who did not exercise belonged to the low-exercise group. Smoking status was categorized into current, past, or never based on their smoking more than 100 cigarettes over their lifetime and smoking during the last 6 months before participating in the study. Alcohol consumption was calculated based on the type, amount, and frequency of alcoholic beverages in the last 6 months. It was calculated by multiplying the drinking frequency by the average alcohol consumed each time.

### MetS definition

The definition of MetS was based on the 2005 revised National Cholesterol Education Program-Adult Treatment Panel III (NCEP-ATP III) criteria modified for Asians reducing the waist circumference (WC) and approved by the Korean Society for the Study of Obesity (KSSO). MetS was defined as the presence of three or more of the following: (1) abdominal obesity (waist circumference ≥90 cm for men and >85 cm for women), (2) low HDL-cholesterol level (<40 mg/dl for men and <50 mg/dL for women); (3) elevated serum triglyceride level (≥150 mg/dl) or current anti-dyslipidemic medication use; (4) elevated fasting blood glucose level (≥100 mg/dl) or current anti-diabetic medication use; (5) elevated blood pressure (average systolic blood pressure >130 mmHg or diastolic blood pressure >85 mmHg) or current blood pressure medication use (16, 17).

### Daily nutrient intake and dietary pattern analysis

The committee of KoGES designed and validated a semi-quantitative food frequency questionnaire (SQFFQ) that included 103 foods commonly consumed by Koreans (18). It estimated the usual Korean daily food intake during the last 6 months. SQFFQ requested information regarding the consumption frequency and amounts of 103 food items with assigned serving sizes. The results were converted into the quantity of intake of 23 nutrients using the computer-aided nutritional analysis program 3.0 developed by the Korean Nutrition Society (KNS) (18).

The 103 food items and alcohol were categorized into 29 pre-combined food groups and used as independent variables in a PCA to find the optimal number of diet factors. The number of factors was assigned according to satisfying eigenvalues of >1.5 in the PCA, which was met in 4 criteria. The orthogonal rotation procedure (Varimax) was applied to generate the clusters, indicating dietary patterns (19). Dietary factor-loading values of ≥0.40 were used to indicate significant contributions of food items by assigning names to the dietary patterns (19). The main food groups of the Korean balanced diet (KBD) included fish, crabs, red meats, vegetables, kimchi, pickles, seaweeds, and mushrooms as main food groups. Noodles, bread, fast foods, soups, and meats were high in the Western-style diet (WSD). A plant-based diet (PBD) included beans, potatoes, green vegetables, seaweed, milk, nuts, and eggs as predominant food groups. Rice-main diet (RMD) was rich in rice.

### Hansik quantification by Korean dietary pattern index (K_diet_-index)

Hansik was quantified based on commonly consumed Korean dishes or foods to determine its effect on MetS. Hansik was defined based on 14 components of traditional Korean meals shown in Table 1, and each component was given the portion size and criteria for a score. Each question about hansik provided portion size and criteria for individual food items to quantify the nutritional status of the participants for their eating as per the hansik pattern. However, the component of “meals with multigrain rice” did not provide the portion size since it indicated the type of the meal, compared to having noodles or bread instead of multigrain rice. Each question was scored 1 if the hansik criteria were satisfied, else it was scored zero. The meal with multigrain rice (a mixture of cooked rice and other grains; jabgogbab) was 1 when the participants had ≥2 meals with multigrain instead of noodles or bread per day. The score of the 14 components was summed, and the summed score was assigned to the K_diet_-index (11). The higher the total K_diet_-index score, the higher the intake per the hansik pattern. The participants were divided into two groups by K_diet_-index score, and they were called the low- (<8) and high-hansik (≥8) groups.

**Table 1:** The definition of hansik (traditional Korean diet) by the K_diet_-index.

	**Portion size**	**Criteria (times)**	**Score**
Meals with multigrain rice		≥2/day	1
Eating frequencies of grains	210 g	≥2/day	1
Eating frequencies of kimchi	50 g	≥2/day	1
Eating frequencies of soup made with fermented soybeans	200 g	≥1/day	1
Eating frequencies of seaweeds	50 g	≥2/day	1
Cooked vegetables with garlic, onions, and ginger	70 g	≥2/day	1
Using frequencies of sesame or perilla oil, among other oils	1 teaspoon	≥50%	1
Eating frequencies of meats including beef, pork, and chicken	60 g	<3/week	1
Eating frequencies of fried foods	60 g	<3/week	1
Eating frequencies of fish or clams	60 g	≥1/day	1
Eating frequencies of soybean products	60 g	≥1/day	1
Eating frequencies of fruits and fruit juicy	200 g	≥2/day	1
Eating frequencies of processed foods	60 g	<1/day	1
Eating nuts	15 g	≥1/day	1

When meeting the criteria, the score of each K_diet_-index component was 1. If not, the score was 0. The K_diet_-index score was calculated with the sum of scores in each component.

### Dietary inflammatory index

The DII was calculated by an equation reported in an earlier study, which was generated based on the dietary inflammatory potential of foods and nutrients having anti- or proinflammatory properties (energy, 32 nutrients, 4 food products, 4 spices, and caffeine). The inflammatory weights of the nutrients were adopted from the earlier report (20). However, some spices, including garlic, ginger, saffron, and turmeric, were omitted from the original equation since their intake was not reported as part of the SQFFQ. The DII calculation was conducted by multiplying the proinflammatory weights of the 38 dietary components by their daily intake and then dividing the sum for each item by 100. The association between DII, K_diet_-index, and MetS risk was calculated with adjusted logistic regression.

### Self-rated health and stress status

Self-rated health (SRH) was determined by the participant's perception of their wellbeing and health. The participants scored their health status from 1 (feeling very healthy) to 5 (feeling very unhealthy). Stress status was evaluated with 18 questions about physical and psychological stress at home and work, and each question was scored from 0 (lowest stress) to 3 (highest stress). The overall stress status scores were estimated by a summation of the scores of the 18 questions. Higher stress status scores indicated the presence of higher stress.

### Statistical analyses

Statistical analyses were carried out using SAS version 9.3 (SAS Institute, Cary, NC, USA). When the sample size was determined using the G^*^Power program with effect size (0.05), power (0.99), and significant level (0.05), the sample size was 1,036. The sample size for each gender was sufficient to satisfy the sample size. The descriptive statistics for categorical variables (e.g., gender and lifestyle) were evaluated based on the frequency distributions of the hansik categories. Chi-squared tests were used to analyze the frequency distributions for categorical variables. Adjusted means and standard errors were calculated for continuous variables based on the hansik categories. The statistical differences between low and high hansik intake were determined using the analysis of covariance (ANCOVA) after adjusting for covariates, including age, residence area, BMI, education, income, energy intake, alcohol drinking, physical exercise, and smoking status. The adjusted odds ratio (ORs) and 95% confidence intervals (CI) for the high-intake of hansik as defined by the K_diet_-index score were determined by multiple regression analysis after covariate adjustment. *P*-values < 0.05 were considered to be statistically significant.

## Results

### Characteristics of the participants according to their gender and hansik intake

The present study included 58,701 participants, 20,293 men, and 38,408 women, who were categorized into low-hansik and high-hansik intake groups based on the criteria of being under or over the K_diet_-index score of 8, respectively. The low-hansik group (<8 hansik scores) included 6,271 men (30.9%) and 7,728 women (69.1%), and the high-hansik group included 14,022 men (20.1%) and 30,680 women (79.9%). More women belonged to the high-hansik group than men (Table 2). The average age of the participants was higher in the high-hansik group than in the low-hansik group. Across both genders, participants were more educated (≥high school) and had lower incomes (<$4,000/month) in the low-hansik group compared to the high-hansik group. A higher proportion of participants exercised regularly and were former smokers in the high-hansik group than in the low-hansik group. The stress scores were much lower in the high-hansik group than in the low-hansik group and lowered in men than women. These results indicated that people with higher hansik intake had lower stress levels. The SRH scores were lower in the high-hansik group than in the low-hansik group, indicating that the participants in the high-hansik group thought themselves healthier than those in the low-hansik group.

**Table 2:** Demographic and sociographic characteristics according to gender and hansik intake defined by the K_diet_-index score.

	**Men (*n* = 20,293)**		**Women (*n* = 38,408)**	
	**Low-hansik (*n* = 6,271)**	**High-hansik (*n* = 14,022)**	**Low-hansik (*n* = 7,728)**	**High-hansik (*n* = 30,680)**
Age (years)	54.1 ± 0.10^b^	57.5 ± 0.07^a^	50.7 ± 0.09^d^	52.8 ± 0.04^c***+++###^
**Education**				
≤Middle school	433 (11.9)	1,320 (14.9)	1,173 (20.3)	5,565 (22.6)
High school	2,773 (76.2)	8,661 (75.4)	4,225 (73.0)	17,646 (71.7)
≥College	432 (11.9)	859 (9.72)^‡‡‡^	388 (6.71)	1,394 (5.67)^‡‡‡^
**Income**				
≤$2,000	407 (6.92)	1,199 (8.92)	739 (10.4)	3,433 (11.8)
$2.000–4,000	2,359 (40.1)	5,848 (43.5)	2,952 (41.7)	13,033 (44.9)
>$4,000	3,112 (52.9)	6,388 (47.6)^‡‡‡^	3,396 (47.9)	12,574 (43.3)^‡‡‡^
Exercise (%)	3,359 (54.1)	8,593 (61.3)^‡‡‡^	3,421 (44.8)	16,603 (54.2)^‡‡‡^
Former smoking	2,424 (39.0)	6,371 (45.5)	112 (1.47)	348 (1.14)
Smoking (%)	2,011 (32.3)	3,653 (26.1)^‡‡‡^	219 (2.87)	530 (1.73)^‡‡‡^
Stress (scores)	14.4 ± 0.15^c^	13.2 ± 0.10^d^	16.1 ± 0.15^a^	15.3 ± 0.07^b***+++^
Self-rated health (scores)	2.58 ± 0.02^b^	2.53 ± 0.01^c^	2.75 ± 0.02^a^	2.73 ± 0.01^a***++#^

The score of each component meeting the criteria was 1. If not, the score was 0. The K_diet_-index score was calculated by summing the scores of each component in the 14 questionnaires. High- and low-hansik intake indicated the K_diet_-index scores ≥8 or <8, respectively. A Higher K_diet_-index score indicated a higher hansik intake. Stress status was estimated with 18 questions about the physical and psychological at home and work, and each question was scored 0 (lowest stress) to 3 (highest stress). The scores of the stress status were estimated with a summation of the scores of 14 questions. The higher scores of the stress status indicated having higher stress.

*** Significant differences by genders at *P* < 0.001.

++ Significant differences by hansik intake at *P* < 0.01,

+++ *P* < 0.001.

# Significant interaction between genders and hansik intake at *P* < 0.05,

### *P* < 0.001.

‡‡‡ Significantly different from the low-hansik group in χ^2^ test in each gender at *P* < 0.001.

a, b, c, d Different superscripts on the values indicated significant differences among the groups in Tukey's test at *p* < 0.05.

### Food intake according to gender and hansik intake

The major food intake in low- and high-hansik was presented in Table 3. Multigrain rice intake was much higher in the high-hansik group than in the low-hansik group. However, white rice, noodles, and bread intakes were much lower in the high-hansik group in both genders (Table 3). Vegetables, fruits, kimchi, and seaweeds exhibited a much higher intake in the high-hansik group than in the low-hansik group in both genders. The adults in the high-hansik group had higher fish intake and lower meat intake than those in the low-hansik group (Table 3). Furthermore, beans, fermented beans, and nuts showed a higher intake in the high-hansik group than in the low-hansik group (Table 3). The results suggested that the 14 components of the hansik definition in Table 1 made the participants well-separated into low- and high-hansik groups.

**Table 3:** Food intake according to gender and hansik intake defined by the K_diet_-index score.

	**Men (*n* = 20,293)**		**Women (*n* = 38,408)**	
	**Low-hansik (*n* = 6,271)**	**High-hansik (*n* = 14,022)**	**Low-hansik (*n* = 7,728)**	**High-hansik (*n* = 30,680)**
Multigrain rice (g/day)	424 ± 5.24^c^	569 ± 3.50^a^	396 ± 5.06^d^	475 ± 2.36 ^b***+++###^
White rice (g/day)	230 ± 4.58^a^	86.7 ± 2.94^c^	148 ± 4.44^b^	50.8 ± 2.05^d***+++###^
Noodles (g/day)	85.6 ± 1.49^a^	57.6 ± 0.96^b^	59.9 ± 1.45^b^	36.8 ± 0.66^c***+++#^
Bread (g/day)	33.3 ± 0.79^a^	29.1 ± 0.51^b^	31.9 ± 0.77^a^	25.3 ± 0.35^c**+++#^
Fruits (g/day)	144 ± 4.36^c^	229 ± 2.81^b^	154 ± 4.24^c^	262 ± 1.92^a***+++##^
Vegetables (g/day)	217 ± 3.50^c^	309 ± 2.25^a^	193 ± 3.40^d^	266 ± 1.54^***+++##^
Kimchi (g/day)	119 ± 2.24^c^	169 ± 1.45^a^	96.9 ± 2.18^d^	131 ± 0.99^b***+++###^
Seaweeds (g/day)	1.32 ± 0.04^d^	2.01 ± 0.03^b^	1.49 ± 0.04^c^	2.28 ± 0.19^a***+++###^
Fish (g/day)	35.3 ± 0.80^c^	45.0 ± 0.51^a^	33.7 ± 0.77^c^	39.5 ± 0.35^b***+++##^
Meats (g/day)	55.5 ± 0.86^a^	46.4 ± 0.55^b^	44.1 ± 0.84^b^	30.2 ± 0.38^c***+++##^
Beans (g/day)	20.8 ± 0.48^b^	28.1 ± 0.31^a^	20.7 ± 0.47^b^	27.5 ± 0.21^a+++^
Fermented soybeans (g/day)	2.97 ± 0.08^c^	4.27 ± 0.05^a^	2.84 ± 0.08^c^	3.95 ± 0.04^b**+++^
Nuts (g/day)	1.07 ± 0.09^d^	1.94 ± 0.06^b^	1.42 ± 0.09^c^	2.46 ± 0.04^a***+++^
KBD (%)	1,886 (30.1)	6,515 (44.3)^‡‡‡^	1,562 (20.2)	9,906 (32.3)^‡‡‡^
PBD (%)	736 (11.7)	3,462 (24.7)^‡‡‡^	1,952 (25.3)	13,426 (43.8)^‡‡‡^
WSD (%)	4,074 (65.0)	6,357 (45.3)^‡‡‡^	3,891 (50.4)	9,230 (30.1)^‡‡‡^
RMD (%)	2,885 (45.7)	3,600 (25.7)^‡‡‡^	3,346 (43.3)	9,760 (31.8)^‡‡‡^
Alcohol (g/day)	32.0 ± 1.22^a^	30.3 ± 0.72^a^	9.68 ± 1.23^b^	8.99 ± 0.49^b***+^

The score of each component meeting the criteria was 1. If not, the score was 0. The K_diet_-index score was calculated by summing the scores of each component in the 14 questionnaires. High- and low-hansik intake indicated the K_diet_-index scores ≥8 or <8, respectively. A Higher K_diet_-index score indicated a higher hansik intake. KBD, Korean-balanced diet; PBD, plant-based diet; WSD, Western-style diet; RMD, rice-main diet.

** Significant differences by genders at *P* < 0.01,

*** *P* < 0.001.

+++ Significant differences by hansik intake at *P* < 0.001.

# Significant interaction between genders and hansik intake at *P* < 0.05,

## at *P* < 0.01,

### *P* < 0.001.

‡‡‡ Significantly different from the low-hansik group in χ^2^ test in each gender at *P* < 0.001.

a, b, c, d Different superscripts on the values indicated significant differences among the groups in Tukey's test at *p* < 0.05.

### Nutrient intake according to gender and hansik intake

The energy intake divided by estimated energy requirement was higher in the high-hansik participants and women (Table 4). An analysis of the percentage of macronutrient intake indicated that carbohydrates and protein intakes were higher, and fat intake was lower in the high-hansik group compared to the low-hansik group in both genders (Table 4). The intakes of fiber, calcium, vitamins C and D, and flavonoids were much higher in the high-hansik group than in the low-hansik group. However, only vitamin C intake met the recommended intake in the high-hansik group in both genders. Sodium intake was higher in the high-hansik group than the low-hansik group in both genders, and its average intake, except the high-hansik in men, was less than the recommended intake (2,300 mg/day) in the American Heart Association (21). Furthermore, potassium intake was also higher in the high-hansik group than in the low-hansik group in both genders. On the other hand, vitamin D and calcium intake did not reach their recommended intake levels. DII scores were lower in the high-hansik group than in the low-hansik group in both genders (Table 4).

**Table 4:** Nutrient intake according to gender and hansik intake defined by the K_diet_-index score.

	**Men (*****n*** = **20,293)**		**Women (*****n*** = **38,408)**	
	**Low-hansik (*n* = 6,271)**	**High-hansik (*n* = 14,022)**	**Low-hansik (*n* = 7,728)**	**High-hansik (*n* = 30,680)**
Energy (EEE %)	86.1 ± 0.42^c^	91.9 ± 0.28^b^	92.8 ± 0.38^b^	101 ± 0.19^a***+++##^
Carbohydrate (En %)	70.9 ± 0.09^c^	71.7 ± 0.06^b^	71.0 ± 0.09^c^	72.1 ± 0.04^a*+++#^
Fat (En %)	14.6 ± 0.07^a^	13.8 ± 0.05^b^	14.5 ± 0.07^a^	13.6 ± 0.03^c***+++^
Protein (En %)	12.8 ± 0.04^c^	13.4 ± 0.02^b^	13.2 ± 0.03^b^	13.6 ± 0.02^a***+++#^
Fiber (g/day)	14.3 ± 0.11^d^	17.2 ± 0.07^b^	12.1 ± 0.10^c^	14.1 ± 0.05^a***+++###^
Calcium (mg/day)	380 ± 2.64^b^	475 ± 1.77^a^	372 ± 2.39^b^	457 ± 1.18^a***+++#^
Potassium (mg/day)	2,034 ± 15.6^c^	2,503 ± 10.4^a^	1,882 ± 15.0^d^	2,299 ± 7.02^b***+++##^
Sodium (mg/day)	2,267 ± 25.0^b^	2,863 ± 16.7^a^	1,918 ± 24.2^c^	2,338 ± 11.3^b***+++###^
Vitamin C (mg/day)	79.6 ± 0.75^b^	113 ± 0.51^a^	80.8 ± 0.68^b^	113 ± 0.34^a+++^
Vitamin D (ug/day)	5.28 ± 0.07^d^	6.49 ± 0.05^b^	5.70 ± 0.06^c^	6.79 ± 0.03^***+++^
DII (scores)	−17.6 ± 1.28^ab^	−26.6 ± 1.02^c^	−14.6 ± 1.64^a^	−20.8 ± 0.85^b**+++^
Total polyphenol (mg/day)	2,308 ± 19.9^c^	2,959 ± 13.3^a^	2,127 ± 19.2^d^	2,566 ± 8.94^b***+++###^
Flavonoids (mg/day)	25.3 ± 0.39^d^	38.9 ± 0.26^b^	28.0 ± 0.36^c^	43.3 ± 0.18^a***+++##^

The score of each component meeting the criteria was 1. If not, the score was 0. The K_diet_-index score was calculated by summing the scores of each component in the 14 questionnaires. High- and low-hansik intake indicated the K_diet_-index scores ≥8 or <8, respectively. A Higher K_diet_-index score indicated a higher hansik intake. EEE, estimated energy requirement; En%, energy percent; DII, dietary inflammation index.

** Significant differences by genders at *P* < 0.01,

*** *P* < 0.001.

+++ Significant differences by hansik intake at *P* < 0.001.

# Significant interaction between genders and hansik intake at *P* < 0.05,

## at *P* < 0.01,

### *P* < 0.001.

a, b, c, d Different superscripts on the values indicated significant differences among the groups in Tukey's test at *p* < 0.05.

When the diet was clustered into 4 dietary patterns, the consumption of a Korean balanced diet (KBD) or a plant-based diet (PBD) was higher, and a Western-style diet (WSD) or a rice-main diet (RMD) was lower in the high-hansik group compared to the low-hansik group. Multigrain rice intake was higher in the high-hansik group than the low-hansik group, and noodle and bread intakes were opposite to multigrain intake (Table 4). It indicated that the Korean meal pattern was changed from a meal with multigrain rice to a meal with flour-based foods such as noodles and bread due to westernization. Alcohol intake was lower in the high-hansik group than in the low-hansik group for both genders (Table 4).

### Scores for individual components of the K_diet_-index to represent hansik intake

The score for “meal with multigrain rice” was lower in the MetS group than in the non-MetS group but only for men, indicating that men in the MetS group ate less rice than those in the non-MetS group. However, the scores for the eating frequencies of grains, kimchi, seaweed, cooked vegetables, meats, and soybean products were significantly higher in women than men. However, there was no significant difference in MetS incidence between the genders (Table 5). The results suggested that though women consumed higher levels of grains, kimchi, seaweed, cooked vegetables, and soybean products and less meat from the hansik components than men, this did not affect the incidence of MetS (Table 5). The score for the eating frequency of soup did not show significant variations by gender or MetS incidence. The eating frequency of fruits and fruit products, processed foods, and nuts were affected by gender and MetS: the scores were higher in women than men and the non-MetS group than in the MetS group (Table 5). The sum of the K_diet_-index scores of each component, called a total K_diet_-index score, was higher in women than men and the non-MetS group than the MetS group, suggesting that women without MetS had a higher hansik intake than the others (Table 5).

**Table 5:** The K_diet_-index score of each component for hansik definition according to gender and metabolic syndrome (MetS).

	**Men (*****n*** = **20,293)**		**Women (*****n*** = **38,408)**	
	**Non-MetS (*n* = 16,695)**	**MetS (*n* =3 ,598)**	**Non-MetS (*n* = 33,706)**	**MetS (*n* = 4,702)**
Meals containing multigrain rice	0.86 ± 0.004^b^	0.81 ± 0.008^c^	0.90 ± 0.003^a^	0.88 ± 0.007^ab***+++##^
Eating frequencies of grains	0.80 ± 0.005^b^	0.81 ± 0.009^b^	0.89 ± 0.003^a^	0.89 ± 0.008^a***^
Eating frequencies of kimchi	0.65 ± 0.006^a^	0.63 ± 0.011^a^	0.54 ± 0.004^b^	0.56 ± 0.010^b***#^
Eating frequencies of soup made with fermented soybeans	0.20 ± 0.005	0.21 ± 0.01	0.19 ± 0.004	0.19 ± 0.009
Eating frequencies of seaweeds	0.19 ± 0.006^b^	0.20 ± 0.01^b^	0.25 ± 0.004^a^	0.25 ± 0.009^a***^
Cooked vegetables with garlic, onions, and ginger	0.20 ± 0.006^b^	0.22 ± 0.01^b^	0.31 ± 0.004^a^	0.31 ± 0.010^a***^
Using frequencies of sesame or perilla oil	0.05 ± 0.003^a^	0.07 ± 0.005^a^	0.03 ± 0.002^b^	0.04 ± 0.005^b***+^
Eating frequencies of meats including beef, pork, and chicken	0.40 ± 0.006^b^	0.39 ± 0.012^b^	0.58 ± 0.004^a^	0.61 ± 0.011^a***^
Eating frequencies of fried foods	0.6222 ± 0.007^b^	0.612 ± 0.012^b^	0.650 ± 0.005^a^	0.621 ± 0.011^b+^
Eating frequencies of fish or clams	0.15 ± 0.005^b^	0.15 ± 0.009^b^	0.20 ± 0.003^a^	0.20 ± 0.008^a***^
Eating frequencies of soybean products	0.19 ± 0.006^b^	0.19 ± 0.010^b^	0.26 ± 0.004^a^	0.25 ± 0.009^a***^
Eating frequencies of fruits and fruit juicy	0.33 ± 0.007^c^	0.31 ± 0.012^c^	0.53 ± 0.005^a^	0.49 ± 0.011^b***+++^
Eating frequencies of processed foods	0.82 ± 0.005^c^	0.82 ± 0.008^c^	0.90 ± 0.003^a^	0.86 ± 0.008^b***+##^
Eating frequencies of nuts	0.16 ± 0.01^c^	0.13 ± 0.01^d^	0.29 ± 0.004^a^	0.24 ± 0.01^b***+++^
Total K_diet_-index score	6.38 ± 0.03^c^	6.34 ± 0.05^c^	6.94 ± 0.02^a^	6.78 ± 0.05^b***++^

The score of each component meeting the criteria was 1. If not, the score was 0. The K_diet_-index score was calculated by summing the scores of each component in the 14 questionnaires. High- and low-hansik intake indicated the K_diet_-index scores ≥8 or <8, respectively. A Higher K_diet_-index score indicated a higher hansik intake. Adjusted means and standard errors were calculated with adjusted for confounding factors such as age, energy intake, body mass index (BMI), residence area, education, income, alcohol intake, smoking status, and physical activity.

*** Significant differences by genders at *P* < 0.001.

+ Significant differences by hansik intake at *P* < 0.05, at

++ *P* < 0.01,

+++ *P* < 0.001.

# Significant interaction between genders and hansik intake at *P* < 0.05,

## at *P* < 0.01.

a, b, c, d Different superscripts on the values indicated significant differences among the groups in Tukey's test at *p* < 0.05.

### Association of the K_diet_-index and DII scores with MetS risk

**Figure 1:**
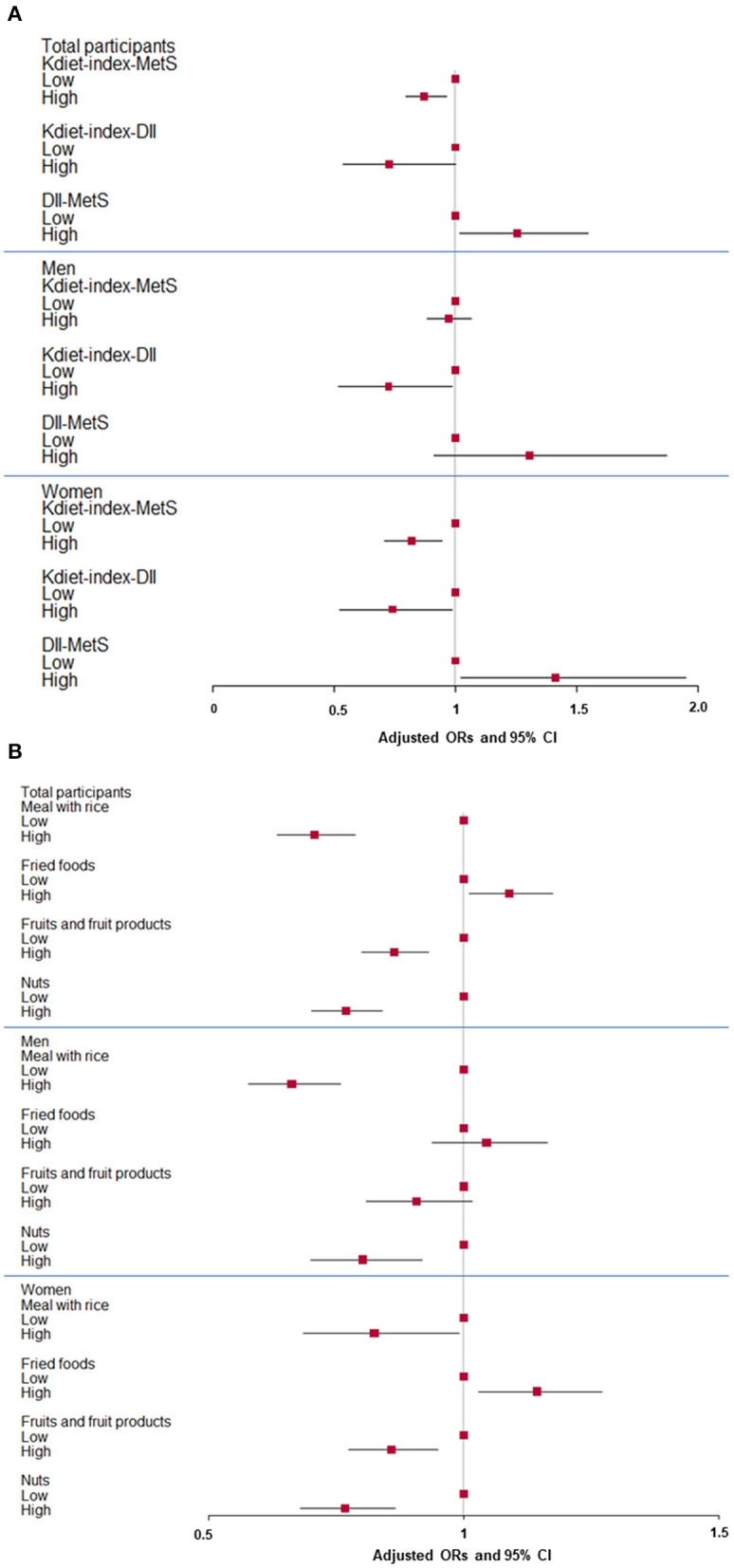
Adjusted odds ratio (ORs) and 95% confidence intervals (CI) of hansik intake evaluated with K_diet_-index score and metabolic syndrome risk (MetS). **(A)** Association of K_diet_-index score with MetS and dietary inflammatory index (DII) and association of MetS and DII in men and women. **(B)** Association of some components of K_diet_-index with MetS in men and women. Hansik was defined with 14 components with portion sizes and scores. The cutoffs of a meal with multigrain rice, fried food intake, fruits, and fruit products, and nut intake were ≥ twice per day, <3 times per week, ≥2 servings per day, and ≥once a day, respectively. Red squares and lines indicated adjusted ORs and 95% CI, respectively. They were shown in total participants, men, and women.

Among all the components of hansik evaluated by the K_diet_-index, it was inversely associated with MetS risk by 0.903 times (95% CI = 0.821–0.993) in total participants and women by 0.817 times (95% CI = 0.707–0.943) but not in men, after adjusting for covariates including age, gender, residence area, education, income, energy intake, exercise, alcohol intake, and smoking status (Figure 1A). K_diet_-index and DII showed an inverse relationship in both genders (*P* < 0.05). DII was positively associated with MetS risk in total participants and women, not in men (*P* < 0.05; Figure 1A). These results suggested that hansik intake could be an anti-inflammatory diet to decrease MetS risk.

In the component of the K_diet_-index, the high intake of a meal with multigrain rice, fruits, and their products, and nuts lowered the risk of MetS 0.707, 0.864, and 0.769 times, respectively, after adjusting for covariates. The low intake of fried foods resulted in a 0.918 time lower risk of MetS in all participants (Figure 1B). The differences in some components were shown in the MetS and non-MetS groups in both genders, but their differences were weaker in men than women. In men, the risk of MetS was 0.663 times lower with the intake of rice as part of the hansik. The scores for processed and fried food intake were higher in the MetS group than in the non-MetS group in women but not in men (Figure 1B), indicating that women with MetS were a lower intake of processed and fried foods. In contrast, in men, the meal with multigrain rice and eating frequencies of nuts were higher in the non-MetS group than in the MetS (Figure 1B). The scores of total K_diet_-index was higher in non-MetS and MetS in both gender. Therefore, people consuming a meal containing rice, fruits, and nuts as a dessert or snack and less processed and fried foods might be less susceptible to the risk of MetS.

### Hansik intake scored by the K_diet_-index and MetS and its components according to gender

The frequency of MetS incidence did not vary significantly between the low-hansik and high-hansik groups. However, the adjusted odds ratio (OR) for MetS was inversely related to high-hansik intake in all participants, especially women (Table 6). In the two-way ANCOVA adjusted for covariates, the body composition indices significantly differed with gender and hansik intake. The BMI, waist circumferences, and fat mass were inversely associated with the high-hansik group, and the skeletal muscle mass index (SMI) was positively associated with the high-hansik group in all participants regardless of gender (Table 6). It indicated that the high-hansik intake was inversely associated with obesity and abdominal obesity. However, glucose metabolism, including fasting serum glucose concentrations, glycosylated hemoglobin (HbA1c), and insulin resistance, did not significantly differ between the low-hansik and high-hansik groups (Table 6). There was no significant association between serum glucose concentration, HbA1c, and insulin resistance in the high-hansik group in both genders. After adjusting for covariates, serum total cholesterol, LDL cholesterol, and triglyceride concentrations were significantly affected by gender and hansik intake scored by the K_diet_-index in all participants (Table 6). Serum concentrations of total cholesterol and LDL cholesterol but not triglyceride were higher in women than men, while they were lower in the high-hansik group than in the low-hansik group in men but not women. Serum HDL cholesterol concentrations were higher in women than men, but they were not significantly different between low- and high-hansik groups (Table 6). Men with a low-hansik intake had higher SBP and DBP, and high-hansik intake was associated with lower SBP and DBP in men. Serum high-sensitivity C-reactive protein (hsCRP) concentrations were not significantly related to the high-hansik group for both genders. However, the estimated glomerular filtration rate (GFR) was much higher in the high-hansik group than in the low-hansik group for both genders, and it was positively associated with high-hansik intake in men, women, and all participants by 1.36, 1.78, and 1.54 times, respectively (Table 6). Serum aspartate aminotransferase (AST) and alanine aminotransferase (ALT) activities were higher in men than women. However, only serum AST activities were higher in the low-hansik group than in the high-hansik group in men. However, there was no association between the high-hansik and low-hansik groups in serum ALT activities (Table 6).

**Table 6:** Characteristics of metabolic parameters according to gender and hansik intake defined by the K_diet_-index score and their associations.

	**Men (*****n*** = **20,293)**			**Women (*****n*** = **38,408)**			**Total**
	**Low-hansik (*n* = 6,271)**	**High-hansik (*n* = 14,022)**	**Adjusted ORs and 95% CI**	**Low-hansik (*n* = 7,728)**	**High-hansik (*n* = 30,680)**	**Adjusted ORs and 95% CI**	**Adjusted ORs and 95% CI**
MetS (%)^1^	1,084 (17.3)	2,514 (18.0)	0.923 (0.843–1.010)	888 (11.5)	3,814 (12.4)	0.876 (0.799–0.961)	0.874 (0.820–0.932)
BMI (mg/kg^2^)^2^	24.6 ± 0.04^a^	24.4 ± 0.03^b^	0.926 (0.863–0.992)	23.6 ± 0.04^c^	23.6 ± 0.02^c***+++##^	0.886 (0.830–0.946)	0.888 (0.847–0.931)
Waist (cm)^3^	86.0 ± 0.11^a^	85.4 ± 0.07^b^	0.911 (0.842–0.985)	78.0 ± 0.10^c^	78.1 ± 0.05^c***++###^	0.886 (0.830–0.946)	0.909 (0.861–0.961)
SMI^4^	7.14 ± 0.01^a^	7.18 ± 0.01^a^	1.912 (1.731–2.113)	6.11 ± 0.01^b^	6.12 ± 0.01^b***^	1.673 (1.530–1.828)	1.759 (1.646–1.879)
Fat mass (%)^5^	23.2 ± 0.07^b^	23.0 ± 0.04^c^	0.899 (0.809–0.999)	30.7 ± 0.07^a^	30.6 ± 0.03^a***+++^	0.866 (0.786–0.955)	0.877 (0.816–0.942)
Serum glucose (mg/dL)^6^	98.6 ± 0.42^a^	98.2 ± 0.27^a^	1.036 (0.918–1.169)	94.0 ± 0.41^b^	93.6 ± 0.18^b***^	0.897 (0.769–1.047)	0.968 (0.880–1.065)
HbA1c (%)^7^	5.72 ± 0.02	5.72 ± 0.01	1.397 (1.121–1.740)	5.71 ± 0.02	5.71 ± 0.01	1.141 (0.885–1.472)	1.214 (0.948–1.500)
Insulin resistance^8^	700 (11.2)	1,612 (11.5)	0.967 (0.831–1.125)	419 (5.42)	1,873 (6.10)	0.866 (0.711–1.056)	0.917 (0.813–1.034)
Serum total cholesterol^9^	195 ± 0.77^b^	189 ± 0.52^c^	0.964 (0.855–1.086)	200 ± 0.74^a^	201 ± 0.35^a***+++###^	0.963 (0.865–1.071)	0.937 (0.866–1.014)
Serum HDL^10^	49.3 ± 0.28^b^	49.3 ± 0.19^b^	0.933 (0.826–1.053)	57.0 ± 0.27^a^	57.3 ± 0.13^a***++^	1.103 (1.001–1.215)	1.039 (0.963–1.120)
Serum LDL^11^	117 ± 0.71^b^	113 ± 0.47^c^	0.978 (0.850–1.125)	121 ± 0.68^a^	120 ± 0.32^a***+++###^	0.943 (0.834–1.066)	0.935 (0.854–1.025)
Serum TG^12^	141 ± 1.77^a^	134 ± 1.18^b^	0.990 (0.899–1.090)	114 ± 1.71^c^	115 ± 0.80^c***+##^	0.970 (0.870–1.083)	0.955 (0.889–1.026)
SBP (mmHg)^13^	127 ± 0.31^a^	125 ± 0.20^b^	0.905 (0.823–0.996)	121 ± 0.30^c^	121 ± 0.14^c***+##^	1.031 (0.930–1.142)	0.947 (0.883–1.015)
DBP (mmHg)^14^	79.2 ± 0.21^a^	78.2 ± 0.14^b^	0.859 (0.750–0.984)	74.1 ± 0.20^c^	74.2 ± 0.09^c***++###^	0.864 (0.729–1.023)	0.849 (0.765–0.944)
Serum hs–CRP^15^	0.16 ± 0.01^a^	0.14 ± 0.01^a^	0.832 (0.592–1.169)	0.13 ± 0.01^ab^	0.13 ± 0.004^b*^	0.903 (0.585–1.393)	0.848 (0.650–1.106)
GFR^16^	81.8 ± 0.33^c^	84.0 ± 0.22^b^	1.363 (1.204–1.543)	84.1 ± 0.32^b^	87.3 ± 0.45^a***+++###^	1.784 (1.572–2.025)	1.535 (1.405–1.678)
Serum AST (U/L)^17^	25.5 ± 0.25^a^	24.9 ± 0.17^b^	0.856 (0.698–1.050)	23.1 ± 0.24^c^	22.9 ± 0.11^c***+^	0.991 (0.763–1.289)	0.897 (0.765–1.053)
Serum ALT(U/L)^18^	26.7 ± 0.37^a^	25.7 ± 0.25^a^	1.010 (0.891–1.144)	19.7 ± 0.36^b^	19.8 ± 0.17^b***#^	0.956 (0.801–1.141)	0.969 (0.875–1.072)

The score of each component meeting the criteria was 1. If not, the score was 0. The K_diet_-index score was calculated by summing the scores of each component in the hansik definition. High- and low-hansik intake indicated the K_diet_-index scores ≥8 or <8, respectively. A Higher K_diet_-index score indicated a higher hansik intake. ORs, Odds ratio; CI, confidence intervals. Covariates for adjustment included age, energy intake, body mass index (BMI), residence area, education, income, alcohol intake, smoking status, and physical activity. The cutoff points of hansik intake for logistic regression were as following:

1 Metabolic syndrome (MetS) criteria;

2 <25 kg/m2 for body mass index (BMI);

3 < 90 cm for men and 85 cm for women waist circumferences;

4 <29.0 % for men and 22.8 % for women in skeletal muscle index (SMI; defined as appendicular skeletal muscle mass/height);

5 < 25% for men and 30% for women for fat mass;

6 <126 ml/dL fasting serum glucose plus diabetic drug intake;

7 <6.5% hbA1c plus diabetic drug intake;

8 <2.54 HOMA-IR;

9 <230 mg/dL serum total cholesterol concentrations;

10 <40 mg/dL for men and 50 mg/dL for women serum HDL cholesterol;

11 <160 mg/dL serum LDL cholesterol concentrations;

12 <150 mg/dL serum triglyceride concentrations;

13 <140 mmHg systolic blood pressure (SBP);

14 diastolic blood pressure (DBP) <90 mmHg plus hypertension medication;

15 <0.5 mg/dL serum high sensitive-C-reactive protein (hs-CRP) concentrations;

16 Estimated glomerular filtration rate (GFR) <60;

17 <40 U/L Aspartate aminotransferase;

18 <35 U/L Alanine aminotransferase.

* Significant differences by genders at *P* < 0.05,

*** *P* < 0.001.

+ Significant differences by hansik intake at *P* < 0.05, at

++ *P* < 0.01,

+++ *P* < 0.001.

# Significant interaction between genders and hansik intake at *P* < 0.05, at

## *P* < 0.01,

### *P* < 0.001.

a, b, c Different superscripts on the values indicated significant differences among the groups in Tukey's test at *P* < 0.05.

## Discussion

The prevalence of MetS increased in Korean men but not women from 2008 to 2017, according to the Korea National Health and Nutrition Examination Survey (KNHANES) (22). It may be related to the differences in diet among men and women. Korean women usually adhere to the hansik diet pattern with multigrain rice, soup, kimchi, and three side dishes. Hansik is known to be high in salt, and there have been diverse opinions about the health benefits of salt intake (23). However, the salts in hansik mainly come from jangs made with solar salts and fermented soybeans, which have been reported to be beneficial for metabolic diseases (24–26). The present study demonstrated that a high meal containing multigrain, fruits, and nuts and a low intake of fried foods based on a K_diet_-index composed of 14 hansik-related components was inversely associated with MetS risk.

Further, the participants with high SRH scores had higher K_diet_-index scores than those with low SRH. women on a high-hansik diet had 0.88 times lower risk of MetS. The high-hansik diet was inversely associated with the MetS components, namely BMI, waist circumference, and fat mass, and positively associated with SMI, an indicator of skeletal muscle mass in both genders. However, among the lipid parameters measured, high hansik intake was inversely associated only with hypo-HDL cholesterolemia. Therefore, hansik intake lowered MetS risk mainly by modulating body composition, particularly in women. Meals with rice might be a primary factor for Asians being leaner than Caucasians.

In Korea, from 2008 to 2017, the MetS prevalence in men increased by 3.6%, but that in women decreased marginally by 1.8%. It may be linked to several lifestyle factors other than diet alone. In the present study, a higher proportion of men belonged to the former and current-smoker group than women, while men and women drank about 31 and 9 g/day of alcohol, respectively. However, men exercised more than women and had lower stress scores. Moreover, men consumed a higher proportion of balanced KBD and WSD than women but less PBD and RMD. Energy (EER %), carbohydrates, and protein intakes were much higher in women than men, but fat intake was higher in men than women in the present study. It is difficult to explain the gender differences in the MetS risk based on lifestyles and nutrient intake. Gender-specific risk factors for MetS have been demonstrated in some studies (27, 28). In two studies in China, a more diversified diet was associated with a lower MetS risk among young women (27), while “animal and fried food” and “high-salt and energy” dietary patterns were observed to be related to a higher risk of MetS in men and women, respectively (28). In the KNHANES, high fruit and juice intakes were lower in the MetS group than in the non-MetS group in men, while breakfast intake was lower in the MetS group than in the health group in women (29). Therefore, the individual components of the diet may influence MetS risk differently for each of the genders.

Previous studies have demonstrated that SRH is influenced by disease status, mental health, nutrient intake, and other risk factors such as smoking and alcohol drinking. A higher SRH is strongly linked to lower mortality risk (30, 31). However, the relationship between SRH and diet quality is inconsistent. In KHANES 2007–2014, SRH was strongly related to a higher intake of nutritious foods such as vegetables and milk in adults (32). However, SRH was not associated with the overall diet quality in adolescent children, although it was positively associated with a high vegetable intake and negatively linked to a high fat intake (33). Therefore, people in Korea consider hansik containing vegetables, fruits, and fish to be a healthy diet; however, they should be taught the right way to consume hansik to prevent metabolic diseases in the adult population.

Hansik is a traditional Korean balanced diet: it includes cooked multigrain rice (jabgogbab), soup (kuk), kimchi, fish, and 2 different vegetables (banchan). Traditional Korean cooking methods are boiling, blanching, seasoning, fermenting, and picking, but frying and baking are not commonly used (7). Kuk is made of jangs instead of salt, with vegetables and meat. Banchan is seasoned with jangs, and herbs, including red pepper, garlic, onion, sesame oil, and perilla oil. The hansik diet pattern was defined using 14 components, and the cutoff of each questionnaire was assigned to describe hansik according to the present study based on the previous studies (7). The Mediterranean diet described by the 14 items exhibits a strong inverse linear association with the adiposity index in adults at high cardiovascular risk. Among the 14 items of the K_diet_-index, a high intake of nuts and a low intake of sweetened/carbonated beverages exhibited the highest inverse relationship with abdominal obesity (34). These results suggest that adherence to the Mediterranean diet prevents MetS risk. Like the Mediterranean diet, a higher intake of hansik (higher total hansik scores) was inversely associated with MetS, especially abdominal obesity-related components in MetS, only in women through low-fat mass and high skeletal muscle mass. Among the 14 components, the scores of meals with rice, eating frequency of fried foods, frequency of consumption of fruits and their products, and eating frequency of processed foods and nuts were significantly different between the non-MetS and the MetS groups only in women in the present study. The sum of 14 hansik component scores was higher in women than men and in the non-MetS group rather than the MetS group. They were highest in the women without MetS. These results indicate that women had better adherence to hansik intake than men and were inversely linked to MetS risk. However, hansik intake was not associated with hyperglycemia in both genders.

Meals with multigrain rice, fish, and vegetables, representing a balanced diet, have been reported to be inversely associated with abdominal obesity in Korea, China, and Japan (35–37), as seen in the present study. Residents of Southern China have traditionally consumed multigrain rice with pork and vegetables and have a lower risk of abdominal obesity (35). Unlike Southern China, people in Northern China traditionally have flour made of wheat, which is positively associated with abdominal obesity (35). Whole grain intake is prospectively and inversely associated with abdominal obesity in adults in the Framingham Offspring cohort and HUNT study (38, 39). However, a white rice-main diet with a lower nutrient score similar to RMD is positively associated with obesity and MetS risk (8, 40). In Korea, a WSD high in noodles, bread, and meats, is positively associated with MetS risk, and KBD alleviated dyslipidemia in RCT (9, 10, 41). Therefore, a Korean-balanced diet with multigrain rice may ameliorate abdominal obesity and MetS risk compared to flour-based meals, including noodles and bread. In the present study, the participants who adhered to the hansik diet pattern had a higher SRH, indicating that people thought a hansik diet would be healthier than a WSD.

Consuming vegetables, seaweed, and fruits is known to reduce MetS risk. Vegetable and fruit intakes are inversely associated with MetS risk (OR: 0.86 and 95% CI: 0.80–0.92 for vegetables; OR: 0.86 and 95% CI = 0.77–0.96 for fruits) in meta-analysis with the studies in Asia (42). However, the meta-analysis with RCTs with MetS patients has shown that vegetables were inversely associated with diastolic blood pressure but no other metabolic syndrome components (43). Seaweed intake (4–6 g/day) is inversely associated with MetS risk in an RCT (44). They are known as healthy foods, but their cooking methods are crucially linked to health. In hansik, raw and cooked vegetables and seaweeds are seasoned with fermented soybeans called jang, perilla oil, and sesame oil in every meal with multigrain rice. Perilla oil and sesame oil include high linoleic acid and linolenic acid, respectively, which are known to prevent and alleviate dyslipidemia and blood pressure (45). Oils can make fat-soluble components in vegetables be absorbed better (46). Therefore, vegetable dishes in hansik are cooked to be healthier.

Furthermore, fruits are used as a dessert instead of sweets. A portion size of fruits is about 2 pieces of seasonal fruits such as apple, pear, persimmon, melon, and tangerine. Their intake of high-hansik (229 + 2.81 g for men and 262 + 262 g for women per day) met the recommended intake from the Korea Nutrition Society and Center for Disease Control and Prevention (2 servings/day; about 200–300 g/day) (47). Nuts have been consumed as a component of snacks and side dishes. Peanuts, walnuts, and dried seeds, including sunflower, sesame, and perilla seeds, have been traditionally used as the components of seasoning and snacks. Nut intake is beneficial for preventing MetS, overweight, and obesity in a meta-analysis of six prospective cohort studies and 62 RCTs (48). In addition, a 13-year population-based prospective study has demonstrated that nuts intake is inversely associated with MetS risk and severity (49). Therefore, the nut intake in adults with high-hansik may have a beneficial impact on reducing MetS risk.

Although hansik is considered a healthy diet in Korea, the impact of its high sodium content on health has been controversial. In the present study, sodium intake was higher in the high-hansik group than the low-hansik group. However, it was lower than the sodium recommended intake (2,500 mg/day) in women (2,338 mg/day) and slightly higher in men (2,863 mg/day). Potassium intake was also higher in the high-hansik than in the low-hansik. Furthermore, GFR was higher in the high-hansik than the low-hansik, suggesting that sodium excretion was better in the high-hansik group than the low-hansik group. Consistent with the GFR result, blood pressure was lower in the high-hansik group than in the low-hansik group in the present study. Consistent with the result, a previous study has shown that MetS is positively associated with urinary sodium and potassium ratio and GFR regardless of sodium intake (50). However, high salt intake is well-known for hypertension and MetS development, and less salt usage in hansik cooking has been promoted in Korean Health Department. Hansik containing less salt would be a better diet for preventing MetS risk.

In addition, hansik has not traditionally included milk and milk products which are critical nutritious factors, especially for bone health and metabolic diseases (29, 51). The intake of milk and milk products is low among Koreans, which is related to their exclusion from the components of hansik. However, participants with high-hansik intake had a higher calcium intake (M: 116 ± 2.77; F: 137 ± 1.29 mL) than those with low-hansik (M: 107 ± 2.87; F: 120 ± 1.92 mL) in the present study. It suggests that the participants with a high-hansik intake had a high SHR score and consumed more healthy foods, including milk and milk products, than those with a low-hansik intake. However, they still did not meet the recommended milk intake levels (52). Thus, efforts should be made to include milk and milk products in hansik meals.

The strength of the present study was that we were able to demonstrate that hansik intake scored by the K_diet_-index was inversely associated with MetS as observed with the Mediterranean diet. However, there were some limitations. First, the results potentially included reverse causality bias due to the use of data from cross-sectional design studies. Second, the measurements for food intake were self-reported and could be over- or underreported: the participants were more likely to report beneficial food intake if they had diseases or were overweight/obese. Therefore, the results might have some potential bias.

## Conclusions

Consuming hansik, consisting of multigrain rice with fruits and nuts, might improve MetS risk, primarily modulating body fat and increasing muscle mass, particularly in women, and blood pressure and dyslipidemia in men. As Koreans believe that consuming a hansik diet improves health status, compliance with the hansik diet pattern might prevent and alleviate MetS risk. However, the hansik diet pattern did not meet the recommended calcium intake levels; hence, milk and milk products should be included as part of the side dishes in the hansik-based diet.

## Data availability statement

The raw data supporting the conclusions of this article will be made available by the authors, without undue reservation.

## Ethics statement

The studies involving human participants were reviewed and approved by the Institutional Review Board (IRB) of the National Institute of Health, Korea (KBP-2015-055) and Hoseo University (HR-034-01). The patients/participants provided their written informed consent to participate in this study.

## Author contributions

HY and SP conceptualized the study. M-SK acquired funding and supervised the research projects. MK, HH, and SP analyzed the data. MK, M-SK, DJ, and SP prepared the manuscript with contributions. All authors approved the final version.

## Funding

This research was supported by the Research Program (E0220602-02) of the Korea Food Research Institute, funded by the Ministry of Science and ICT.

## Conflict of interest

The authors declare that the research was conducted in the absence of any commercial or financial relationships that could be construed as a potential conflict of interest.

## Publisher's note

All claims expressed in this article are solely those of the authors and do not necessarily represent those of their affiliated organizations, or those of the publisher, the editors and the reviewers. Any product that may be evaluated in this article, or claim that may be made by its manufacturer, is not guaranteed or endorsed by the publisher.
